# Clinico-Epidemiological Profile of Children Orphaned due to AIDS Residing in Care Giving Institutions in Coastal South India

**DOI:** 10.1155/2019/4712908

**Published:** 2019-11-03

**Authors:** Rekha Thapar, Meher Singha, Nithin Kumar, Prasanna Mithra, Bhaskaran Unnikrishnan, Ramesh Holla, Vaman Kulkarni, B. B. Darshan, Avinash Kumar

**Affiliations:** ^1^Department of Community Medicine, Kasturba Medical College, Mangalore, Faculty of Health Sciences, Manipal Academy of Higher Education, Manipal, India; ^2^Kasturba Medical College, Mangalore, Manipal Academy of Higher Education, Manipal, India

## Abstract

**Background:**

HIV/AIDS has a greater impact on children. Besides being orphaned by the untimely demise of one or both parents due to the disease, these children are more prone for discrimination by the society.

**Methods:**

In this cross-sectional study 86 children orphaned by AIDS residing in care giving institutions for HIV positive children in Mangalore were assessed for their clinico-epidemiological profile and nutritional status. Institutional Ethics Committee clearance was obtained before the commencement of the study. The collected data were analyzed using SPSS (Statistical Package for Social Sciences) version 11.5 and the results expressed in mean (standard deviation) and proportions. BMI was calculated and nutritional status assessed using WHO Z scores (BMI for Age) for children between 5 and 19 years separately for boys and girls.

**Results:**

The mean age of the children was 13.2 ± 3 years. Majority (*n* = 56, 65.1%) of the children were double orphans. Most of the children orphaned by AIDS (*n* = 78, 90.7%) had a history of both the parents being HIV positive. The median CD4 count of participants at the time of our study was 853.5 (IQR 552–1092) cells/microliter. A higher percentage of orphans were malnourished compared to nonorphans. (41.1% vs. 36.7%). All the educational institutions, wherein the children orphaned by AIDS were enrolled, were aware about their HIV status. Five of the participants felt discriminated in their schools. Only two of the participants felt discriminated by their friends because of their HIV status.

**Conclusion:**

From our study we draw conclusion that even though the children orphaned due to AIDS are rehabilitated in terms of having shelter and provision of education and health care, much needs to be done in terms of improving the nutritional status of these children and alleviating the discriminatory attitude of the society towards them.

## 1. Introduction

Even after 38 years since the very first case of AIDS was reported [1], HIV/AIDS remains a global public health challenge assuming pandemic proportions. It consumes vast amount of resources in terms of cost incurred on treatment as well as amount of years lost in terms of DALYs (Disability Adjusted Life-Years). Initially considered to be a disease affecting men and women in their reproductive age group, the disease has greater impact on children [2].

The annual incidence of HIV infection among children less than 15 years has reduced drastically between the years 2010 and 2015. In 2015, an estimated 1.8 million (1.5–2.0 million) children aged 0–14 years were living with HIV, while 150 000 (110 000–190 000) children were newly infected, and 110 000 (84 000–130 000) children had died of AIDS-related causes [3]. Although the adult HIV prevalence has declined throughout the world, the number of children affected by or made vulnerable to HIV remains alarmingly high [4].

Globally 13.4 million children and adolescents (0–17 years) have been orphaned due to AIDS, with Sub-Saharan Africa accounting for 80% of these children [5]. India accounts for 12% of all new HIV infections with an estimated 0.06 million children < 15 years of age infected with HIV/AIDS [6]. However, there are still many children who remain undiagnosed or are diagnosed at a very late stage into the disease. Many of the diagnosed children are not put on Antiretroviral therapy (ART) because they lack access to it. Among the children exposed to HIV in 21 high burden countries in the year 2015, only 54% of them were tested for the disease. In 2016, only 43% of the 1.8 million children living with HIV had access to ART [7].

The term “orphans due to AIDS” or “children orphaned by AIDS” is used to refer children who have lost at least one parent due to the disease [5]. Besides being orphaned due to the untimely demise of one or both parents due to HIV/AIDS, these children are psychologically and socially burdened along with increased morbidities. In the early stage of the disease, children orphaned by AIDS are usually looked after by their surviving parent or grandparents or immediate relatives. With the progression of the disease, many of the children are left to fend for themselves, while others are placed in institutional care. Residential care or institutional care refers to group living arrangements where care is provided by paid adults who would otherwise not be regarded as traditional care givers in that society [8].

Children orphaned by AIDS are more prone to stigmatization and discrimination by their friends, relatives, and society. These children may take to the streets after being abandoned by their own family. These children are susceptible to various forms of abuse—physical, psychological, mental, and sexual. They are also vulnerable to malnutrition and infections owing to the lack of access to food, education, and health care. Children in general are more prone to morbidities in the form of infections like Acute Respiratory Infections (ARI), Acute diarrheal disease (ADD), and other communicable diseases. The susceptibility to these infections is increased in children with HIV/AIDS due to their weakened immune status.

In many instances the decision of placing the HIV infected child under institutional care is taken without considering the general wellbeing of the child, but the circumstances or conditions in the family. Many of the children are placed in institutions by their care givers who consider them to be a burden, ignoring the fact that these children are deprived of their parental bond at a very early age and are emotionally and psychologically fragile. The problems faced by these children continue to exist in the four walls of the institutional care. Even though their basic physical and health needs are taken care to some extent, it is the lack of emotional support that drives these children towards vulnerability. There is limited literature describing the status of children orphaned by HIV/AIDS placed in care giving institutions. The present study was carried out with the objective of assessing the clinico-epidemiological profile and nutritional status of children orphaned by AIDS residing in different care giving institutions in Mangalore.

## 2. Materials and Methods

### 2.1. Background Information of the Study Area

Mangalore is the chief port city and district headquarters of Dakshina Kannada District in the Indian state of Karnataka. With a literacy rate of 93.7% [9] it is one of the fastest growing cities in India in terms of education and provision of health care services. The adult HIV prevalence (15–49 years) in Karnataka is 0.47% (0.37%–0.63%) which is higher than the National average of 0.22% [6]. With around 2.47 lakhs people estimated to be living with HIV, Karnataka is the third highest state with PLHIV [6]. The estimated number of children less than 15 years living with HIV in the state is 12346 [10].

### 2.2. Methods

This cross-sectional study was conducted among 86 children orphaned by AIDS residing in care giving institutions for HIV positive children in Mangalore. There are four care giving institutions for HIV positive children in Mangalore which are all NonGovernment Organizations (NGOs). All the care giving centres provide multitude of services which include basic services like food, clothing and shelter, medical services like treatment for opportunistic infections, ART, CD4 testing, counselling, and palliative care. Vocational training and support for PLHIV is also provided at these centres. The institutions were approached and two of them gave their approval to conduct the study.

Clearance was obtained from Institutional Ethics Committee of Kasturba Medical College, Mangalore to conduct the study. The nature and purpose of the study were explained to the administration of the institutions. All the children residing in these two institutions during the study period were included. The study was conducted over a period of two months. The children were interviewed after obtaining their assent and informed consent from the administration of the institutions. Interview was conducted using a predesigned, semistructured questionnaire consisting of sections on socio demography and HIV profile of the children and their parents, clinical profile of the children, and self-reported stigma and discrimination. Section on stigma and discrimination was filled exclusively for school going children. The interview was conducted in a separate room and took around 15 minutes for each interview. The clinical profile of the children was documented from the case sheets available in the care giving institutions. Weight was measured to the nearest 500 grams and height to the nearest 0.1 cm. Details of the children who were not able to provide answers were taken from the caregivers.

### 2.3. Data Analysis

The obtained data were entered in and analyzed using SPSS (Statistical Package for Social Sciences) version 11.5. The results are expressed in mean (standard deviation) and proportions. BMI was calculated and nutritional status assessed using WHO Z scores (BMI for Age) for children between 5 and 19 years separately for boys and girls [11]. In our study, children who had lost both their parents were termed double orphans. The term maternal orphan was used to describe children who had lost their mother and paternal orphans for those who had lost their father. For purpose of analysis, the children were categorized as orphans and nonorphans. Children who had lost both their parents were categorized as orphans. The rest of the children were grouped under nonorphans. The association between the nutritional status and orphan—hood was assessed using Chi square test, and a *P* value <0.05 was considered statistically significant.

## 3. Results

A total of 86 children orphaned by AIDS living in HIV care giving institutions in Mangalore were included in our study. The study consisted of 56 (65.1%) orphans and 30 (34.9%) nonorphans. The mean age of the children was 13.2 ± 3 years with higher proportion of children in the age group between 10 and 15 years. Majority of the children orphaned by AIDS (*n* = 53, 61.6%) in these institutions were males. Eighty-four children were enrolled in schools with more than half (*n* = 45, 52.3%) attending private educational institutions. Two children did not attend schools due to physical impairments. (One blind and other deaf mute) Socio-demographic characteristics of the study participants are given in Table 1.

The HIV profile of parents of the children orphaned by AIDS is depicted in Table 2. Majority (*n* = 56, 65.1%) of the children were double orphans. Most of the children orphaned by AIDS (*n* = 78, 90.7%) had a history of both the parents being HIV positive. Only 11 children orphaned by AIDS, i.e., 12.8% had other family members positive for HIV.

The clinical profile of the children orphaned by AIDS is shown in Table 3. The median CD4 count of participants at the time of our study was 853.5 (IQR 552–1092) cells/microliter. Majority (*n* = 70, 81.4%) of the participants had CD4 counts of ≥ 500 cells/microliter. Seventy-four children were currently on ART out of whom 52 (60.5%) were on ART since the last 5 years. One child was tested sputum positive for Tuberculosis and was undergoing treatment under DOTS (Directly Observed Short Course Treatment).

A higher percentage of orphans were malnourished compared to nonorphans. (41.1% vs. 36.7%). No significant association was observed between nutritional status and orphanhood (*P* > 0.05) as depicted in Table 4.

Discrimination as experienced by the children orphaned by AIDS is shown in Table 5. All the educational institutions wherein the children orphaned by AIDS were enrolled, were aware of their HIV status. However, five of the participants felt discriminated in their schools. Among the 81 (96.4%) participants who had friends outside the care giving centres, only 32 (39.5%) of them disclosed that their friends were aware of their HIV status. Only 2 of the participants felt discriminated by their friends because of their HIV status.

## 4. Discussion

Children living with HIV/AIDS are the worst group affected by the global HIV epidemic. This is evident not only in terms of increased morbidity and mortality among the children but also with an increase in the number of children orphaned due to AIDS.

Many of these orphans are accommodated in different care giving institutions—Government as well as NonGovernmental Organizations (NGOs). However, with the rise in number of children orphaned due to AIDS, these institutions are overburdened in terms of lack of trained manpower and infrastructure. This in turn leads to difficulty in providing better care to these children. The current study was conducted to assess the clinico-epidemiological profile of children orphaned by AIDS living in care giving institutions in Mangalore. In our study, majority of children orphaned by AIDS were males. A study by United States Agency for International Development (USAID) which included three HIV care giving NGOs in India reported a greater number of male orphans [12]. Similar observations were made in primary studies in orphanages in rural China [13], and India [14] where the percentage of male orphans was higher in number. In contrast, other similar studies in India [15, 16], Ethiopia [17], and China [18] reported a greater number of female orphans compared to males.

In most of the HIV prevalent countries, the absence of parental care is combined with scarcity of a proper system for care of children orphaned due to AIDS. This has led to lack of access to basic education and health care among these children. In our study, it was heartening to see that all the orphans were enrolled in school and have access to ART. Education instills important life skills into children which makes them self-sufficient in their adult life, more so in children with HIV/AIDS where it lowers the risk of spread of HIV and improves nutrition. Education has been rightly considered a “Social Vaccine” against HIV/AIDS [19]. In this regard school plays an important role where students can acquire information regarding HIV/AIDS and its prevention.

United Nations International Children Emergency Fund (UNICEF) in its report from highly AIDS affected countries states that orphans are more likely to attend schools than nonorphans [19]. United States Agency for International Development (USAID) in High HIV-prevalence countries in Sub-Saharan Africa also reported that orphan hood had varied effect on school attendance. Higher school attendance was reported for orphans in some African countries except in Zimbabwe where there was no difference. The report also stated that maternal orphans faced difficulty in completing primary school as compared to nonorphans [20]. Enrollment in educational institutions was higher in our study with 97.7% children attending schools. The percentage of children orphaned by AIDS enrolled in schools was 70% in a study in Ahmedabad [15] and 91.5% in Ethiopia [17].

The prevalence of HIV/AIDS is high in India and most of the African countries where heterosexual route is the most common route of transmission. In these countries, the probability of HIV infected person transmitting it to the other partner is very high. This in turn means their children are at a higher risk of losing both the parents and them becoming double orphans, compared to nonHIV orphans. Most of the children orphaned by AIDS in our study had a history of both the parents being HIV positive. (*n* = 78, 90.7%). Double orphans are the most vulnerable among the children orphaned by AIDS since these children are devoid of love and care from both their parents. In our study, 65% of the children were double orphans. having lost both their parents, whereas the proportion of double orphans from a report compiled by USAID in orphanages in India was around 37% [12].

One of the important factors deciding the treatment of HIV/AIDS is the CD4 count. Treatment in an HIV infected especially children results in a reduced risk of opportunistic infection as well as improved CD4 count. All our study participants were on ART. The median CD4 count of participants at the time of our study was 853.5 (IQR 552–1092) cells/microliter. The median CD4 count was higher among the orphan group compared to nonorphans. In contrast a study conducted in Asia reported a higher median CD4 count among the nonorphans as compared to the orphans [21]. The finding in our study could be due to a smaller number of nonorphans. In a study in Hyderabad the mean absolute CD4 count was 1058.97 cells per microliter [16].

Children living under institutional care, especially those who are HIV positive have a greater risk of being poorly nourished as compared to children not residing in institutions [8]. These poorly nourished children are more at risk of contracting infections, and subsequently dying because of it. A study done in Malawi substantiates the findings that children in orphanages were more likely to be undernourished compared to nonorphans [22]. A higher percentage of orphans in our study were malnourished compared to nonorphans. In a study done in South India [14], the percentage of undernourishment in institutionalized orphans was 72%, whereas in a study in Ahmedabad [15] 20% of the children were found to be malnourished. In a study in Asia, most study participants who had low weight for age Z scores were orphans [21]. In a study in Hyderabad, India, 59.7% children were stunted, 46.8% were underweight, and 19.5% had low BMI for age [16]. Studies have also shown a relationship between CD4 counts and the nutritional status of the children reporting that malnutrition weakens the immune response to ART there by creating a time gap where the child is still prone for opportunistic infections (OIs) [23].

HIV discrimination refers to unfair and unjust treatment of someone based on their real or perceived HIV status. These discriminations are often based on ignorance and misconceptions about the transmission of HIV, sexual behavior of people, drug use and fear of contracting the disease [24]. Discrimination may result in social isolation of the child, loneliness, lack of interest in studies. low self-esteem amongst the children, and absence of social security.

In our study, all the educational institutions wherein the children orphaned by AIDS were enrolled, were aware of their HIV status. In a study in Ahmedabad, 37% of the study subjects', parents, friends, and teachers were aware of the HIV status [15].

Stigma and discrimination due to HIV/AIDS has become a part of this global pandemic and has emerged as one of the greatest obstacles for prevention and control of HIV/AIDS. The discriminatory attitude towards HIV infected, which initially arose due to the uncertainties surrounding the disease, mode of transmission, coupled with its high prevalence among high risk groups, continues to persist in our society. Children infected with HIV who are innocent victims of this disease have not been spared by this discriminatory and prejudiced attitude of our society. They are marginalized by their relatives who refuse to take care of these children owing to their HIV status. Some of these children are also refused admission in the schools. Those who are attending schools may have to discontinue due to the discriminatory attitude of the teachers and classmates. Discrimination by their friends and classmates is the reflection of the attitude of the parents who do not want their wards to mingle with children infected with HIV/AIDS. The HIV status is not disclosed in the schools or to their friends by the immediate care taker or by the children themselves due to the fear of discrimination.

In our study, five of the participants felt discriminated in their schools. In a study in Ethiopia [17], 6.7% felt discriminated by their friends. In a study from nine Southern African countries on HIV related discrimination among school going children, the prevalence of discrimination was between 10% and 20% [25]. Among the 81 (96.4%) participants in our study, who had friends outside the care giving centres, only 32 (39.5%) of them disclosed that their friends were aware of their HIV status. Only 2 of the participants in our study felt discriminated by their friends because of their HIV status.

## 5. Conclusion

From our study as well as studies from HIV care giving institutions in different parts of the world, we draw conclusion that even though these children are rehabilitated in terms of having a home to stay and are provided education and health care, much needs to be done in terms of improving the nutritional status of these children and alleviating the discriminatory attitude of the society towards them.

The limitation of the study would be that findings from two HIV care giving institutions cannot be generalized to all the care giving institutions in the country. The age of children, the type of facilities available and care provided might vary from region to region. It also depends upon whether these centres are Government funded or run by an NGO. Whatever be the context, the fact remains that the children orphaned due to AIDS are God's own children who must bear the double burden of being orphans as wells as being HIV positive.

With the rise in the number of children orphaned due to AIDS, there is a need to increase the number of HIV care giving institutions. However, providing these children a place to stay is not the end point. Capacity building of all the HIV care giving institutions to provide holistic care and rehabilitative medical, nutritional, and educational services to children orphaned due to AIDS should be carried out. Focus group discussions should be carried out to identify the problems faced by the children. Care givers of these institutions should also be included in these focused discussions to identify the barriers faced by them in providing care to these children. At a programmatic level, these problems identified should translate into policies to safeguard the rights of these children, alleviate the stigma and discriminatory attitude towards them, thus providing them a better future.

## Figures and Tables

**Table 1:** Socio-demographic characteristics of the study participants [*N* = 86].

Characteristics	Orphans *N* = 56	Nonorphans *N* = 30	Total *N* = 86
*Mean age ± SD years*	13.4 ± 3.1	12.7 ± 2.7	13.2 ± 3
*Age in years*	*n* (%)	*n* (%)	*n* (%)
<10 years	07 (12.5)	06 (20.0)	13 (15.1)
10–15 years	37 (66.1)	19 (63.3)	56 (65.1)
>15 years	12 (21.4)	05 (16.7)	17 (19.8)

*Gender*			
Male	42 (75.0)	11 (36.7)	53 (61.6)
Female	14 (25.0)	19 (63.3)	33 (38.4)

*Place of origin*			
Mangalore	15 (26.8)	12 (40.0)	27 (31.4)
Outside Mangalore	41 (73.2)	18 (60.0)	59 (68.6)

*Enrolled in school/college*			
Yes	55 (98.2)	29 (96.7)	84 (97.7)
No	01 (01.8)	01 (03.3)	02 (02.3)

*Type of school/college*			
Private	27 (48.2)	18 (60.0)	45 (52.3)
Government	28 (50.0)	11 (36.7)	39 (45.3)
Do not attend school	01 (01.8)	01 (03.3)	02 (02.3)

**Table 2:** HIV profile of parents of study participants [*N* = 86].

HIV profile		*n* (%)
Living status of parents	Double orphans	56 (65.1)
	Maternal orphans	14 (16.3)
	Both parents alive	11 (12.8)
	Paternal orphans	05 (05.8)

HIV Status of parents	Both positive	78 (90.7)
	Status unknown	06 (07.0)
	Only mother positive	01 (01.2)
	Both negative	01 (01.2)

HIV status of other family members	No sibling-all other members negative	61 (70.9)
	Sibling negative	14 (16.3)
	Sibling positive	08 (09.3)
	No sibling-other family member positive	03 (03.5)

**Table 3:** Clinical Profile of study participants [*N* = 86].

Variables	Orphans *N* = 56	Nonorphans *N* = 30	Total *N* = 86
Median CD4 count (IQR)	872.50 (545–1035)	848.50 (560.25–1228.75)	853.50 (552–1092)
*CD4 count (cells/microliter) * ^∗^	*n* (%)	*n* (%)	*n* (%)
<200	02 (03.6)	01 (03.3)	03 (03.5)
200–499	09 (16.1)	04 (13.3)	13 (15.1)
≥500	45 (80.4)	25 (83.3)	70 (81.4)
*Currently on HAART*			
Yes	49 (87.5)	25 (83.3)	74 (86.0)
No	07 (12.5)	05 (16.7)	12 (14.0)
*Duration of treatment* [*N* = 74]			
Less than 1 year	03 (05.4)	01 (03.3)	04 (04.7)
1–5 years	16 (28.6)	14 (46.7)	30 (34.9)
More than 5 years	37 (66.1)	15 (50.0)	52 (60.5)
*Mode of Transmission*			
Vertical	56 (100)	30 (100)	86 (100)
*Immunization status*			
Completely immunized for age	56 (100)	30 (100)	86 (100)

^∗^At the time of study.

**Table 4:** Nutritional status of the study participants [*N* = 84].

Weight for height *Z* scores	Orphans (*N* = 56)	Nonorphans (*N* = 30)	Chi-square *P* value
	*N* (%)	*N* (%)	
Normal (Median ± 2 SD)	33 (58.9)	19 (63.3)	0.159*P* > 0.05
Median ± 3 SD	23 (41.1)	11 (36.7)	

**Table 5:** Self-reported discrimination as experienced by the study participants [*N* = 84].

Characteristics		Orphans	Nonorphans	Total^∗^
		*n* (%)	*n* (%)	*n* (%)
Discrimination faced in school (*N* = 84)	Yes	03 (05.4)	02 (06.9)	05 (05.9)
	No	52 (94.6)	27 (93.1)	79 (94.1)
Have friends outside the care centres (*N* = 84)	Yes	53 (96.4)	28 (96.6)	81 (96.4)
	No	02 (03.6)	01 (03.4)	03 (03.6)
Friends aware of HIV status (*N* = 81)	Yes	17 (31.5)	15 (55.6)	32 (39.5)
	No	37 (68.5)	12 (44.4)	49 (60.5)
Discrimination by friends (*N* = 81)	Yes	02 (03.7)	00 (00.0)	02 (02.5)
	No	52 (96.3)	27 (100.0)	79 (97.5)

^∗^Only school/college going children orphaned by AIDS were included.

## Data Availability

The data used to support the findings of this study are available from the corresponding upon request.
